# Spectrum of Pure Red Cell Aplasia in a Tertiary Care Hospital in Northeast India

**DOI:** 10.7759/cureus.79364

**Published:** 2025-02-20

**Authors:** Biswajit Dey, Vandana Raphael, Darilin M Shangpliang, Yookarin Khonglah, Jaya Mishra, Evarisalin Marbaniang, Nirvana Thangjam

**Affiliations:** 1 Pathology, North Eastern Indira Gandhi Regional Institute of Health and Medical Sciences (NEIGRIHMS), Shillong, IND

**Keywords:** bone marrow, diamond-blackfan anemia, erythroblast, pure red cell aplasia (prca), reticulocytopenia

## Abstract

Background

Pure red cell aplasia (PRCA) is an uncommon disorder characterized by severe normocytic normochromic anemia, reticulocytopenia, and an absence of erythroblasts from otherwise normal bone marrow. PRCA can present as either a congenital disorder or an acquired syndrome.

Methodology

A total of six cases of PRCA diagnosed in a tertiary care hospital from January 2016 to December 2019 were retrospectively studied. The diagnosis of PRCA was made based on bone marrow examinations showing erythroblastopenia with essentially normal myelopoiesis and megakaryopoiesis.

Results

Of the six patients, one was identified as having congenital PRCA, three were classified as having primary acquired PRCA, and two were diagnosed with secondary acquired PRCA. The congenital case was an infant diagnosed with Diamond-Blackfan anemia (DBA). There were three cases of primary acquired PRCA. One was idiopathic, as no specific cause could be identified. The other primary acquired case had autoimmune hemolytic anemia (AIHA); however, no underlying cause for peripheral hemolysis or secondary causes of marrow suppression could be identified. The third primary case was myelodysplastic PRCA. The causes of secondary acquired PRCA included systemic lupus erythematosus (SLE) and thymoma.

Conclusion

The present study demonstrates that the causes and outcomes of PRCA are varied; however, they are similar to those observed in other similar series.

## Introduction

Pure red cell aplasia (PRCA) is a rare hematopoietic disorder characterized by progressive normocytic normochromic anemia of sudden onset, reticulocytopenia (absolute reticulocyte count of <10,000/µL or <1%), and an almost complete absence of erythroid precursors in the bone marrow [1]. The platelet count, leukocyte count, and leukocyte differentials are characteristically normal [2]. The hemoglobin level decreases rapidly, at approximately 0.1 g/dL per day, corresponding to the red blood cell lifespan; hence, these patients eventually become transfusion-dependent [3].

The causes of PRCA are varied and are broadly divided into congenital and acquired forms [4]. The congenital form is known as Diamond-Blackfan anemia (DBA), typically seen in infancy and early childhood [5]. The acquired form may be primary or secondary to another disorder or agent. Primary PRCA is either idiopathic, where the cause is unknown, or primary immune-mediated such as erythroblastopenia of childhood [4]. Myelodysplastic syndrome (MDS) can also present with the morphologic appearance of primary acquired PRCA. Secondary acquired causes include infections (bacterial or viral), drugs (such as erythropoietin and carbamazepine), collagen vascular disorders (such as systemic lupus erythematosus (SLE) and rheumatoid arthritis), solid tumors (such as thymoma), post-ABO-incompatible stem cell transplant, and various other hematologic conditions [5,6]. PRCA is a disease with significant variability in its clinical presentation, pathological characteristics, and underlying etiology [4-6].

PRCA is a rare disorder. Limited data and information regarding the underlying causes, clinical presentations, and outcomes of this condition are available. Therefore, the present study was undertaken to retrospectively review and describe the clinical presentations, causes, and outcomes of PRCA cases diagnosed in a tertiary care hospital.

## Materials and methods

A retrospective review was conducted of all patients diagnosed with pure red cell aplasia (PRCA) at a tertiary care hospital from January 2016 to December 2019. The study included six patients diagnosed with PRCA during this period. Clinical details, laboratory findings, and relevant imaging studies were collected from patient records.

For each patient, the following investigations were reviewed: complete blood hemogram, bone marrow examination, serum markers for antinuclear antibodies (ANA), direct Coombs test, erythropoietin levels, and, in cases of suspected thymoma, chest computed tomography (CT) scans. Serological tests for B19 parvovirus, human immunodeficiency virus (HIV), hepatitis B virus (HBV), and hepatitis C virus (HCV) were also reviewed, but none of the cases were positive for these viruses.

Complete blood count, peripheral blood smear, and bone marrow examination reports of the cases were collected. All the cases had erythroblastopenia with normal myeloid cells and megakaryocytes, alongside a reticulocyte count of less than 1%. All cases had normal platelet and leukocyte counts. The results of flow cytometry and fluorescence in situ hybridization (FISH) wherever done were collected. The patients' clinical presentations, including symptoms and underlying comorbidities, were also carefully collected.

The patients were categorized into congenital and acquired PRCA. The acquired cases were further classified into primary (idiopathic or antibody-mediated) and secondary acquired PRCA, based on the underlying causes identified through further diagnostic testing.

## Results

Six patients were diagnosed with PRCA between January 2016 and December 2019. One was an infant diagnosed with the congenital form of PRCA (Diamond-Blackfan anemia), three were identified as having primary acquired PRCA, and the remaining two were diagnosed with secondary acquired PRCA.

The congenital case was an infant diagnosed with Diamond-Blackfan anemia. The diagnosis of DBA was made since the patient had presented with facial dysmorphism, anemia, reticulocytopenia, high fetal hemoglobin, and a reduction in red cell precursors in the bone marrow. The one primary acquired PRCA case was idiopathic, as no specific cause could be identified. Although the other primary acquired case had autoimmune hemolytic anemia (AIHA), no underlying cause for peripheral hemolysis or secondary causes of marrow suppression could be identified. Somatic mutation analysis was not done for either case. Primary MDS PRCA was diagnosed based on marrow erythroblastopenia and dysplasia, negative intrinsic marrow pathology on flow cytometry, and 5q deletion on FISH. The causes of secondary acquired PRCA in the present cohort included systemic lupus erythematosus and thymoma.

**Figure 1:**
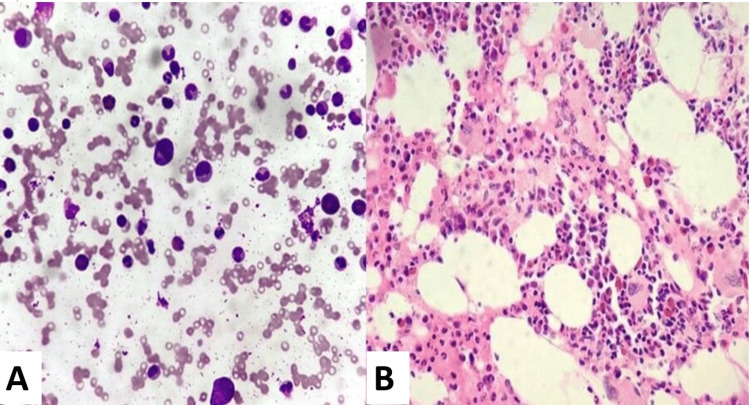
(A and B) Bone marrow aspirate and biopsy showing suppressed erythropoiesis with normal granulopoiesis and megakaryopoiesis (Leishman stain: 400×)

The age of the five patients with acquired PRCA ranged from 24 to 82 years with a median age of 51 years. Total leukocyte and platelet counts were within normal limits for all patients. Reticulocytopenia was observed in all patients ranging from 0.1% to 0.5%. Bone marrow aspirate smears revealed a paucity of erythroblasts ranging from 1% to 8% with a mean count of 4.2% (Figure 1A, 1B). The clinical and laboratory findings for the six patients with pure red cell aplasia are presented in Table 1.

**Table 1:** Clinical and investigation parameters of the patients with pure red cell aplasia AIHA, autoimmune hemolytic anemia; ANA, antinuclear antibody; CECT, contrast-enhanced computed tomography; dsDNA, double-stranded deoxyribonucleic acid; EPO, erythropoietin; FC, flow cytometry; FISH, fluorescence in situ hybridization; Hb, hemoglobin; M/E, myeloid-to-erythroid ratio; PBS, peripheral blood smear; Retic, reticulocyte; RBCs, red blood cells; SLE, systemic lupus erythematosus; MDS, myelodysplastic syndrome; WBC, white blood count; M, male; F, female

Patient number	Age/gender	Complaints	Examination	Hb (g/dL)	Retic (%)	WBC (×10^3^)	Platelet (×10^3^)	Bone marrow	Other tests	Diagnosis
1	7 months/M	Fever	Pallor and facial dysmorphism	4.6	0.3	8.1	200	Normocellular M/E > 40:1	ANA and Coombs test: negative	Diamond-Blackfan anemia
2	30/M	Fatigue and giddiness	Hepatosplenomegaly	7.2	0.1	9.0	490	Normocellular M/E > 10:1	ANA and Coombs test: negative	Primary idiopathic
3	54/M	Fatigue	Pallor and hepatomegaly	3.0	0.3	5.2	195	Normocellular M/E > 10:1	Coombs test, positive; ANA, negative; PBS, fragmented RBCs; FC, negative	Primary acquired (with AIHA)
4	82/M	Generalized weakness	Pallor and hepatomegaly	3.4	0.2	4.93	231	Hypercellular M/E > 20:1	EPO, 31.5 mU/mL; FC, negative; FISH, del(5q)	Primary MDS
5	24/F	Generalized weakness and joint pain	Pallor	4.3	0.5	6.1	180	Diluted smears	ANA and dsDNA: positive	SLE
6	51/M	Breathlessness and palpitations	Pallor	5.6	0.5	8.7	345	Normocellular M/E > 25:1	CECT chest: mediastinal mass	Thymoma

**Figure 2:**
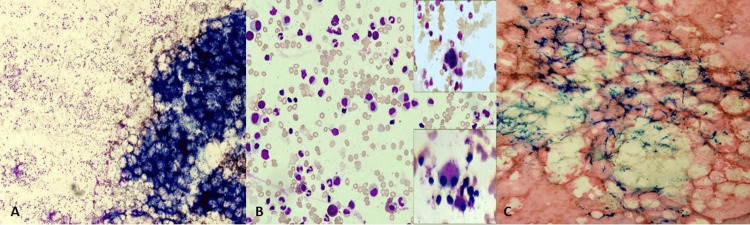
(A) BMA showing hypercellular marrow with diluted cell trails (Leishman stain: 100×). (B) BMA with hypogranular myelocytes and suppressed erythropoiesis (Leishman stain: 400×). Inset: Dysplastic megakaryocytes, multinucleated and binucleated megakaryocyte (Leishman stain: 400×). (C) Perls stain (100×) showing an increase in iron stores BMA: bone marrow aspirate

**Figure 3:**
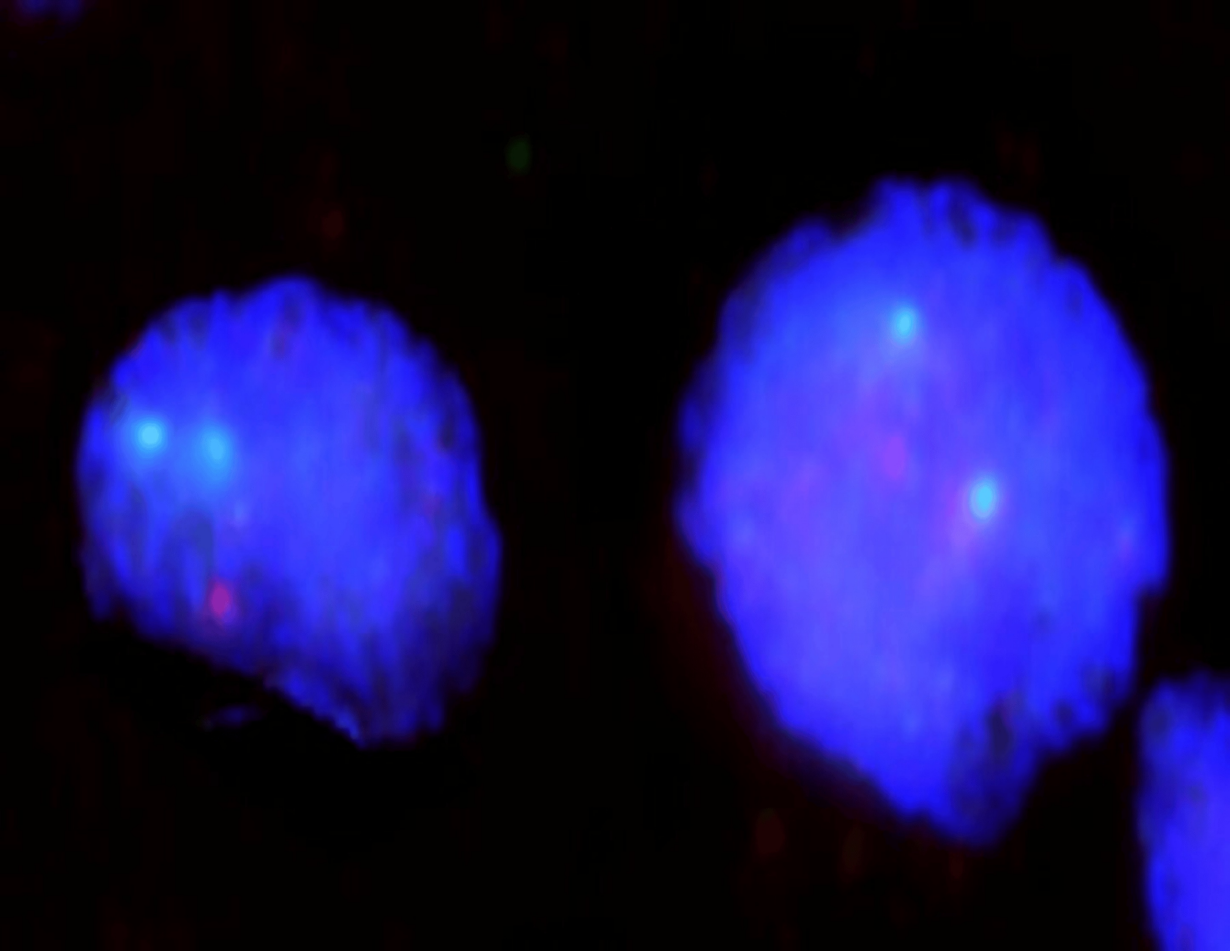
Interphase nuclei with a signal constellation indicative of a deletion in 5q, i.e., two green signals and one orange signal

Patient 1, diagnosed with the congenital form of PRCA (Diamond-Blackfan anemia), responded to prednisolone but did not maintain follow-up. Patients 2, 3, and 4 were diagnosed with primary acquired PRCA. Since no cause could be identified despite thorough laboratory investigations, patient 2 was considered idiopathic. He showed a good response to prednisolone, with the remission of the anemia. Patient 3 had AIHA, confirmed through peripheral blood examination and laboratory investigations, including a positive direct Coombs test; no underlying cause could be elicited. However, this patient did not maintain follow-up. Patient 4, an elderly man, had bone marrow features suggestive of dyspoiesis (Figure 2A, 2B, 2C) and 5q deletion on FISH (Figure 3). He was diagnosed with primary MDS PRCA. He was given supportive therapy but passed away three months later following hospitalization.

Patient 5 presented with generalized weakness and joint pain and was found to have a positive antinuclear antibody (ANA) test and anti-double-stranded DNA (anti-dsDNA), confirming a diagnosis of systemic lupus erythematosus (SLE). She was treated with nonsteroidal anti-inflammatory drugs and prednisolone, showing a good response after six months of follow-up. Patient 6 was diagnosed with thymoma type B based on a computed tomography (CT) of the chest and histopathology. However, he did not undergo a thymectomy and expired five months later due to congestive cardiac failure.

## Discussion

Kaznelson first described PRCA in 1922 [1]. It is a condition typically diagnosed after other causes of anemia have been considered and excluded [7]. PRCA can occur in individuals aged 7-70 years [1]. The congenital form of the disease is usually detected within the first 18 months of life. It is often associated with congenital abnormalities, including exophthalmos, strabismus, pterygium colli, bilateral double ureters, hydronephrosis, abnormal osseous development, and congenital heart lesions. This form of the disease is frequently responsive to corticosteroids [8].

The acquired form of PRCA may be primary or secondary to various conditions. A thorough research of the primary form of the disease has identified factors that may contribute to the absence of erythroblasts in the bone marrow and has led to the development of new treatment approaches for this condition [8]. A notable characteristic of primary acquired PRCA is its strong association with various immunologic abnormalities, where an immune mechanism disrupts erythroid differentiation [5]. Although the exact pathogenesis is not fully understood, it is believed to be mediated by humoral antibodies, natural killer (NK) cells, or T-cell-mediated damage to erythroid precursors. Humoral and cytotoxic immune responses mediate PRCA associated with autoimmune hemolytic anemia. Corticosteroids were the first immunosuppressive drugs used to treat autoimmune-associated PRCA and remain the treatment of choice, particularly in young adults [9]. Myelodysplastic primary acquired PRCA is an uncommon manifestation of myelodysplasia, characterized morphologically by erythroid hypoplasia; however, its pathophysiology is distinct from that of other types of PRCA [5].

A variety of factors have been reported as causes of secondary acquired PRCA, including infections such as B19 parvovirus, drugs, toxins, solid tumors such as thymoma, autoimmune and collagen vascular disorders such as systemic lupus erythematosus (SLE) and rheumatoid arthritis, lymphoproliferative disorders particularly chronic lymphocytic leukemia, and a range of other conditions [5]. Thymoma is considered the most common cause of secondary PRCA, with the highest incidence rate (13.21%), followed by B19 parvovirus-associated PRCA (11.32%) [10]. Patients with thymoma typically require surgical resection, and the remission of PRCA has been reported in 25%-30% of patients who undergo thymectomy [11]. Human B19 parvovirus is the most common viral cause of transient or reversible PRCA [4]. B19 parvovirus causes PRCA by exerting a cytotoxic effect on early erythroid progenitor cells after binding to the P antigen on the red cell surface [4]. In immunocompromised patients, B19 parvovirus can lead to chronic PRCA and pancytopenia due to the suppression of hematopoiesis in early marrow progenitor cells [4,12]. A high index of clinical suspicion and a battery of laboratory investigations are required to diagnose and categorize a case of PRCA [2,13].

Studies show that the majority of the cases are acquired. Among the acquired causes, most cases in the adult group were secondary PRCA, while primary PRCA is more common in the pediatric group [14-18]. A comparison with other Indian studies is summarized in Table 2.

**Table 2:** Comparison with other similar Indian studies on PRCA PRCA: pure red cell aplasia

Authors (year)	Duration of study	Study population	Number of cases	Most common cause of PRCA
Marwaha et al. (2002) [14]	11 years	Pediatric	16	Congenital
Srinivas et al. (2007) [15]	10 years	Both pediatric and adult	39	Idiopathic (acquired)
Malhotra et al. (2008) [16]	3 years	Adult	9	Idiopathic (acquired)
Kala et al. (2022) [17]	4 years	Both pediatric and adult	9	Acquired
Kurhade et al. (2023) [18]	6 years	Pediatric	11	Acquired
Present study (2025)	4 years	Both pediatric and adult	6	Acquired

The goal of the treatment of PRCA is to induce remission and restore erythropoiesis, thereby reducing the need for blood transfusions and minimizing transfusion-related complications [19]. The primary objective is to address and remove the underlying cause. If anemia does not improve, corticosteroids are considered first-line therapy [7]. To date, no universal consensus has been established for second-line therapy, which may include androgens (e.g., danazol), immunosuppressants (e.g., cyclosporine A and cyclophosphamide), immunoglobulins, immunotherapy (e.g., rituximab), and procedures such as splenectomy and stem cell transplantation [11].

Limitations of the study

This study has several limitations, including the small sample size of only six patients, which limits generalizability. Being retrospective, the accuracy of patient records and data may be incomplete. Some of the cases do require additional workup such as somatic mutation analysis for confirmation but were not done due to financial constraints. The findings are from a single center, potentially not reflecting the broader population. Larger, multicenter studies are needed for more comprehensive insights into PRCA.

## Conclusions

PRCA is a rare hematologic disorder that can present in both congenital and acquired forms, with a wide range of etiologies. This study highlights the diverse causes of acquired PRCA, including idiopathic, autoimmune-related, and secondary causes such as thymoma and SLE. Our findings demonstrate that PRCA can affect individuals across a broad age range, and a thorough diagnostic workup, including bone marrow examination and the exclusion of other causes of anemia, is essential for accurate diagnosis. The response to treatment varies depending on the underlying cause.
